# Decreased circulating dipeptidyl peptidase-4 enzyme activity is prognostic for severe outcomes in COVID-19 inpatients

**DOI:** 10.2217/bmm-2021-0717

**Published:** 2022-02-23

**Authors:** Ákos Nádasdi, György Sinkovits, Ilona Bobek, Botond Lakatos, Zsolt Förhécz, Zita Z Prohászka, Marienn Réti, Miklós Arató, Gellért Cseh, Tamás Masszi, Béla Merkely, Péter Ferdinandy, István Vályi-Nagy, Zoltán Prohászka, Gábor Firneisz

**Affiliations:** 1 Ramgen Plc., Budapest, H-1126, Hungary; 2 Department of Internal Medicine & Haematology, Research Laboratory, Semmelweis University, Budapest, H-1088, Hungary; 3 Department of Anaesthesiology & Intensive Care Medicine, Central Hospital of Southern Pest, National Institute of Haematology & Infectious Diseases, Budapest, H-1097, Hungary; 4 Department of Infectology, Central Hospital of Southern Pest, National Institute of Haematology & Infectious Diseases, Budapest, H-1097, Hungary; 5 Department of Internal Medicine & Haematology, Semmelweis University, Budapest, H-1088, Hungary; 6 Department of Haematology & Stem Cell Transplantation, Central Hospital of Southern Pest, National Institute of Haematology & Infectious Diseases, Budapest, H-1097, Hungary; 7 Department of Pharmacology & Pharmacotherapy, Semmelweis University, Budapest, H-1089, Hungary; 8 Semmelweis University, Budapest, H-1085, Hungary; 9 Central Hospital of Southern Pest, National Institute of Haematology & Infectious Diseases, Budapest, H-1097, Hungary

**Keywords:** circulating DPP4 activity, COVID-19, disease severity, DPP4, mortality, prognostic biomarker, SARS-CoV-2, T2DM

## Abstract

**Aim:**

To investigate the serum circulating DPP4 activity in patients with COVID-19 disease.

**Materials & methods:**

Serum samples from 102 hospitalized COVID-19 patients and 43 post-COVID-19 plasma donors and 39 SARS-CoV-2 naive controls and their medical data were used. Circulating DPP4 activities according to different COVID-19 disease peak severity (WHO) groups at sampling and at peak were assessed.

**Results:**

A significant decrease (p < 0.0001) in serum DPP4 activity was found in study groups of higher disease severity. When the circulating DPP4 activity was assessed as a prognostic marker, the logistic regression (p = 0.0023) indicated that the enzyme activity is a predictor of mortality (median 9.5 days before death) with receiver operating characteristic area under the curves of 73.33% (p_[area = 0.5]_ < 0.0001) as single predictor and 83.45% (p_[area = 0.5]_ < 0.0001) in combination with age among hospitalized patients with COVID-19.

**Conclusion:**

Decreased circulating DPP4 activity is associated with severe COVID-19 disease and is a strong prognostic biomarker of mortality.

COVID-19 is an infectious disease caused by SARS-CoV-2 [1]. In contrast to prior emerging coronaviruses, such as MERS-CoV and SARS-CoV, the total number of deaths associated with COVID-19 is higher, but the case fatality rate is lower [2]. Severe illness occurred in 15.7% of patients after admission to a hospital in the initial phase of the COVID-19 outbreak [1].

The spike (S) glycoprotein of MERS-CoV targets DPP4. A putative receptor-binding domain (RBD) on the viral S protein that mediates the interaction with DPP4 as a receptor is critical for viral binding and entry into the target cell [3]. DPP4 was also suggested as a receptor of SARS-CoV-2 using bioinformatic approaches [4,5]. Furthermore, flexible molecular docking simulations predicted that hydrogen bonding and salt bridge interactions could occur between specific residues of the RBD of SARS-CoV-2 and DPP4; however, the interactions were predicted to be weaker than with MERS-CoV [6]. In contrast, subsequent direct experiments revealed that SARS-CoV-2 did not bind human DPP4 [7]. In addition, a variant promoter region of the *DPP4* gene inherited from Neandertals was reported to double the risk of becoming critical ill with COVID-19 [8].

Interestingly, multiple analyses from independent retrospective observational studies reported significant improvement in severe outcomes and mortality among patients with COVID-19 and type 2 diabetes mellitus (T2DM) using a DPP4 inhibitor [9,10,11]. The inactivating cleavage of incretin hormones by DPP4 made it an attractive drug target in the treatment of T2DM. DPP4 has a membrane-bound form (also known as CD26) and a soluble form, with detectable enzymatic activity in human serum [12]. Because non-alcoholic fatty liver disease (NAFLD) is highly prevalent in obesity and T2DM, which are established risk factors for severe clinical COVID-19 disease course [13], and higher serum DPP4 enzymatic activity has been reported in patients with NAFLD [14], we hypothesized that circulating DPP4 activity might be altered in patients with acute COVID-19 disease and DPP4 might have prognostic value.

## Materials & methods

### Study design & participants

The authors conducted a non-interventional, observational, retrospective cohort study, with 184 Hungarian adult participants. Hospitalized patients with acute SARS-CoV-2 infection (acute COVID-19 disease, n = 102) and those recovered from prior SARS-CoV-2 infection and in the convalescent phase (when donating plasma for the treatment of other patients with COVID-19 disease, n = 43) were enrolled in the study from 16 April to 2 July 2020 at two institutions in Budapest (Semmelweis University and South Pest Central Hospital – National Institute of Hematology and Infectious Diseases). In addition, a group of ‘non-COVID-19 controls’ (n = 39) were employed with available results on the determined serum DPP4 activity from an ongoing metabolic study of adult females. These blood samples were all taken before September 2019, prior to possible exposure to SARS-CoV-2 in Hungary.

Subsequent determination of the serum DPP4 enzymatic activity in the serum samples originating from the above detailed collections was performed by Ramgen, Budapest. The clinical data, including the COVID-19 disease outcomes, were blinded for Ramgen until the circulating DPP4 activities were measured in the serum samples and the results were not reported to the clinical centers. Only subsequently were the anonymized clinical data of the participants shared with Ramgen, which subjected all the data to a thorough analysis and assessed the relationship specifically between circulating DPP4 activity and COVID-19 disease and its prognosis.

All the serum samples transferred to Ramgen originated from study participants who had signed informed consent for the whole project and all the sample collections. The subsequent study on serum DPP4 activity conducted by Ramgen possessed the ethical approval of the national ethical body of Hungary (Medical Research Council Scientific and Research Committee, reference: IV/4403-4/2020/EKU; 30 December 2020). The study was conducted according to the Declaration of Helsinki.

The following prespecified criteria were applied for this study.

#### Inclusion criteria

- Age >18 years (adult, older adult);
- Both sexes were eligible;
- The participant or his/her legally authorized representative signed and provided informed consent;
- Either confirmed SARS-CoV-2 infection by nasopharyngeal swab PCR in hospitalized patients (‘acute COVID-19' study group) during an ongoing acute COVID-19 disease or prior to the sampling in individuals recovered from COVID-19 disease (‘plasma donors’) or;
- Sampling before September 2019 (SARS-CoV-2 non-exposed study group);
- Access to routine clinical records, including laboratory results, drug use and COVID-19 disease outcomes.

#### Exclusion criteria

- The patient or his/her legal representative is unable to provide informed consent;
- Use of a DPP4 inhibitor within 7 days of sampling for circulating DPP4 activity measurement;
- Active tuberculosis or latent tuberculosis infection with <3 months of enrollment;
- Heart failure or volume overload as the principal cause of bilateral pulmonary 'infiltrates' (edema);
- Acute myocardial infarction;
- Absolute neutrophil count <0.6 g/l;
- Pregnancy.

#### Outcome measures

- Serum DPP4 activity (all participants);
- Clinical outcomes of patients with acute COVID-19 disease;
- Routine demographic data, laboratory data and drug use (all participants).

### Procedures

**Figure 1.**
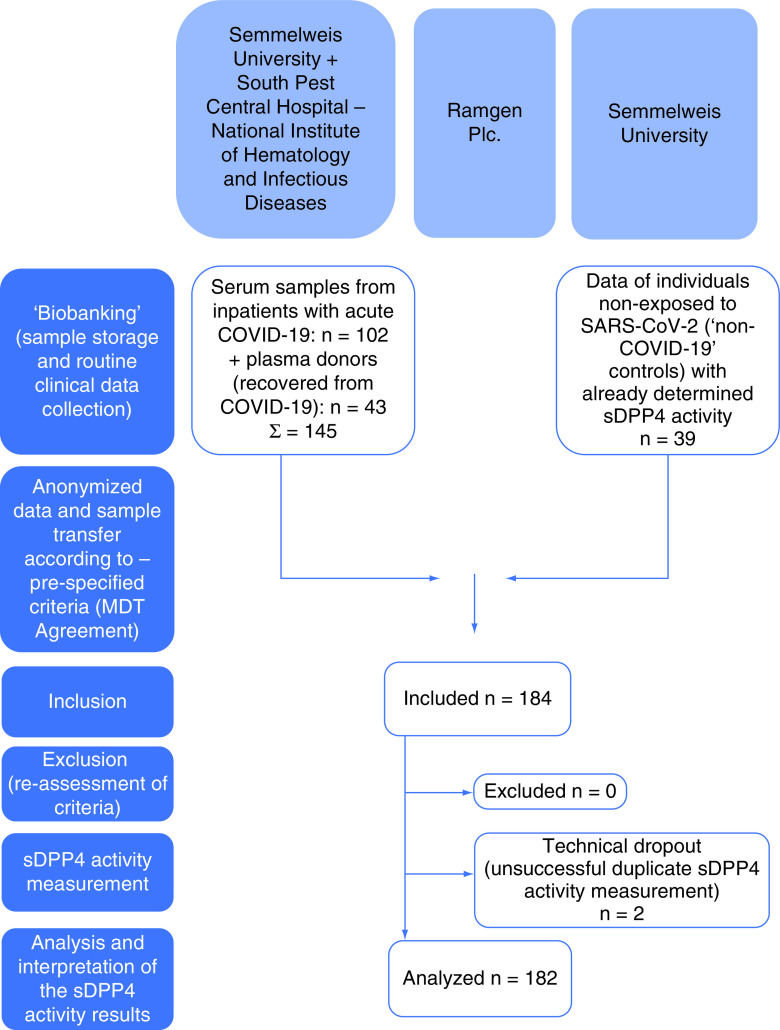
A patient flow diagram.

At baseline, eligibility and medical history, including medical drug use, were assessed and informed consent was obtained from all participants. Blood sampling of patients with acute COVID-19 disease was performed during their hospital stay, before or at maximal COVID-19 disease severity but rarely and not uniformly at admission. After separation of serum, the samples were stored at -80°C at Semmelweis University, and the remaining samples from an earlier, unconnected research project [15] and anonymized clinical data were transferred to Ramgen on the day of the circulating DPP4 activity measurement (summarized on the flowchart in Figure 1). The risk of obtaining poor clinical data as the most frequently observed bias in retrospective studies was minimized, resulting in a good-quality clinical data transfer.

The study participants were classified into the following groups stratified by SARS-COV-2 infection status and COVID-19 disease outcome; peak disease severity was defined on the WHO ordinal scale [16]:

0: never exposed to SARS-CoV-2 (all participants in the 'non-COVID-19' group provided samples before September 2019 and were females undergoing a 75 g oral glucose tolerance test [OGTT] in another study) (n = 39)

1–2: in convalescent phase, recovered from acute SARS-CoV-2 infection (plasma donors) – 1: no prior hospitalization due to COVID-19 (n = 26), 2: prior hospitalization due to COVID-19 (n = 17), 3–6: hospitalized and with acute COVID-19, 3: not requiring O_2_ therapy (n = 27; WHO ordinal scale: 3), 4: required O_2_ therapy via nasal cannula only (n = 33; WHO ordinal scale: 4), 5: intensive care unit (ICU) admission required (due to invasive mechanical ventilation or other cause) and survived (n = 17; WHO ordinal scale: 6 + 7), 6: died in COVID-19 (n = 25; WHO ordinal scale: 8). Neither high-flow oxygen nor non-invasive ventilation therapy was extensively used in the participating study centers during the sample collection period, and therefore no patient was classified into WHO ordinal scale 5.

Serum DPP4 activity was determined in a continuous monitoring assay using the BioTekELx808 (Agilent, CA, USA) microplate reader at 405 nm (with background subtraction), 37°C for 30 min, using 9.4 μl of serum and 115.6 μl of assay buffer (100 mM Tris-HCl, pH 7.6) containing 2 mmol/l H-Gly-Pro-paranitroanilide*p-tosylate substrate (Bachem, Bubendorf, Switzerland) in each microplate well. All samples were measured in duplicates, the factor calculation method was used and reported circulating DPP4 activity was calculated as the mean of two corresponding measurements and expressed in nmol/ml/min (U/l) of pNA hydrolyzed as described [17]. Assays, employing Gly-Pro-pNA substrate to monitor DPP4 activity, have already been used in randomized, controlled clinical trials with pharmacological inhibitors of DPP4 [18]. The serum DPP4 measurement was technically unsuccessful in the case of two participants whose clinical data were used to characterize the study population, but not the DPP4 results.

### Statistical analysis

Data distributions (including circulating DPP4 activities) were assessed using the Shapiro–Wilk test. Differences in central tendencies among groups (multiple comparisons) were assessed using the Kruskal–Wallis test (as the distribution of DPP4 data was non-normal).

After *a priori* ordering (of serum DPP4 activities), the Jonckheere–Terpstra trend test was used to assess an ordered alternative hypothesis (i.e., to assess whether the trend was significant) when it had more statistical power than the Kruskal–Wallis test. The differences in central tendencies between two groups were tested using the Mann–Whitney U test (non-normal). To predict a single binary outcome (such as mortality in COVID-19) using independent prognostic variables (e.g., the circulating DPP4 activity), the logit model (binomial logistic regression) was applied. Multinomial logistic regression was used to predict the nominal dependent variables (the COVID-19 disease outcomes) using one or more independent prognostic variables. The Spearman rank-order (SRO) test was used to assess correlations when data were non-normally distributed. To evaluate the diagnostic/prognostic ability of a test (DPP4 activity measurement), the receiver operating characteristic (ROC) curve was analyzed. The area under the ROC (AUROC) curve was used as a general measure of prognostic accuracy. Data analysis was done using TIBCO Statistica software (version: 13.4.0.1.) and 'R' program (version: 4.0.3). A univariate logistic regression model (circulating DPP4 activity) was also built to predict the probability of death in hospitalized patients with acute SARS-CoV-2 infection and was subsequently adjusted to the patients' ages and other important clinical risk factors from their medical history, as well as to the most important initial laboratory prognostic candidates known from the literature [19,20] (19 separate multivariate logistic regression models).

## Results

The participants' clinical characteristics, including medical history and laboratory data, are indicated in Table 1. The routine laboratory values of hospitalized patients with acute SARS-CoV-2 infection are indicative for the time point when the sampling for the serum DPP4 measurement was performed.

**Table 1.** Clinical characteristics and routine laboratory data of enrolled participants with acute or prior severe acute respiratory syndrome coronavirus 2 infection (‘COVID-19’ patients) and non-exposed controls.

Clinical parameter	Recovered from acute SARS-CoV-2 infection (plasma donors, n = 43)		Hospitalized and with acute SARS-CoV-2 infection (n = 102)				p-value for multiple comparison	Non-COVID-19 control group
	Not requiring hospitalization due to COVID-19 (n = 26)	Required hospitalization due to COVID-19 (n = 17)	Not requiring oxygen therapy (WHO scale: 3; n = 27)	Oxygen via nasal cannula only (WHO scale: 4; n = 33)	ICU care (due to invasive mechanical ventilation or other cause) and survived (WHO scale: 6 + 7; n = 17)	Hospitalized due to COVID-19 and deceased (WHO scale: 8; n = 25)		
Age (years)	45.0 (34.0–54.0)	50.0 (43.0–62.0)	57.0 (42.0–69.0)	67.0 (63.0–78.0)	59.0 (50.0–68.0)	76.0 (72.0–80.0)	<0.0001	37.0 (34.0–40.0)
Sex (male/female)	(15/11)	(12/5)	(17/10)	(20/13)	(8/11)	(11/14)	0.52^†^	(0/39)
Diabetes mellitus (any type)	1/26 (3.86%)	2/17 (11.76%)	4/27 (14.81%)	8/33 (24.24%)	2/17 (11.76%)	11/25 (44%)	0.024^†^	1/39 (2.56%)^§^
Hypertension	7/26 (26.92%)	5/17 (29.41%)	13/27 (48.15%)	22/33 (66.67%)	11/17 (64.71%)	20/25 (80%)	0.0013^†^	2/39 (5.13%)
Chronic heart disease	0/26 (0%)	1/17 (5.88%)	6/27 (22.22%)	14/33 (42.42%)	3/17 (17.65%)	11/25 (44%)	0.045^†^	0/39 (0%)
Chronic pulmonary disease	0/26 (0%)	2/17 (11.76%)	3/27 (11.11%)	6/33 (18.18%)	4/17 (23.53%)	9/25 (36%)	0.22^†^	0/39 (0%)
Chronic liver disease	NA	NA	4/25 (16%)	1/28 (3.57%)	0/14 (0%)	1/23 (4.35%)	0.23^†^	14/39 (35.9%)^‡^
Dementia or other CNS disease	NA	NA	0/25 (0%) 4/25 (16%)	3/28 (10.71%) 7/28 (25%)	1/14 (7.14%) 2/14 (14.29)	6/23 (26.09%) 9/23 (39.13%)	0.24^†^	0/39 (0%)
Malignancy	0/26 (0%)	0/17 (0%)	4/26 (15.38%)	2/32 (6.25%)	8/17 (47.06%)	9/25 (36%)	0.0095^†^	1/39 (2.56%)
White blood cell count (g/l)	6.61 (5.5–7.41)	5.22 (4.83–6.65)	6.25 (4.85–7.42)	6.22 (4.98–7.69)	6.55 (4.82–7.76)	7.45 (4.78–11.58)	0.4	6.62 (5.56–7.48)
Absolute lymphocyte count (g/l)	1.99 (1.84–2.4)	1.95 (1.72–2.25)	1.58 (0.99–2.18)	1.52 (0.96–1.85)	0.91 (0.8–1.25)	0.77 (0.5–1.1)	<0.0001	1.75 (1.56–2.23)
D-dimer (ng/ml)	207.5 (158.0–453.0)	289.0 (209.0–449.0)	1460.0 (610.0–2210.0)	851.0 (530.0–1526.0)	1658.0 (912.5–3080.0)	1430.0 (1106.0–4380.0)	<0.0001	NA
Fasting blood glucose (mmol/l)	NA	NA	4.7 (4.2–5.9)	4.7 (4.35–5.35)	5.86 (4.64–6.2)	6.1 (4.7–8.1)	0.09	5.2 (4.9–5.4)
ASAT (U/l)	NA	NA	21.0 (18.0–31.0)	31.0 (22.0–42.0)	40.0 (28.0–60.0)	51.0 (30.0–82.0)	0.0001	21.5 (19.0–25.0)
ALAT (U/l)	NA	NA	19.0 (13.0–20.0)	27.0 (.0–42.0)	35.0 (26.0–65.0)	34.5 (14.5–71.0)	0.01	18.0 (14.0–23.0)
gGT (U/l)	NA	NA	30.5 (23.5–47.0)	64.0 (24.0–71.0)	100.0 (25.0–144.0)	72.0 (26.0–92.0)	0.29	19.0 (14.0–27.0)
Alkaline phosphatase (U/l)	NA	NA	75.5 (56.0–90.0)	76.0 (56.0–96.0)	85.5 (59.5–158.0)	106.0 (71.0–143.5)	0.07	76.0 (60.0–98.0)
Serum albumin (g/l)	48.0 (47.0–50.0)	46.0 (44.5–47.5)	35.0 (32.8–37.3)	33.0 (26.8–38.1)	32.0 (26.4–37.0)	29.15 (24.6–34.5)	<0.0001	46.1 (43.2–48.4)
Creatinine (μmol/l)	NA	NA	76.0 (51.0–94.0)	70.0 (59.0–85.0)	72.0 (53.0–104.0)	139.0 (62.0–189.0)	0.07	64.0 (58.0–70.0)
CRP (mg/l)	1.0 (0.3–2.5)	0.45 (0.2–0.95)	11.95 (5.6–41.0)	36.8 (17.5–88.6)	111.0 (61.3–169.1)	149.1 (54.9–196.8)	<0.0001	3.5 (1.2–9.9)
PCT (ng/ml)	NA	NA	0.03 (0.02–0.06)	0.02 (0.02–0.14)	0.17 (0.06–0.26)	0.34 (0.11–1.11)	<0.0001	NA
Ferritin (ng/ml)	NA	231.0 (224.0–238.0)	320.0 (163.0–547.0)	379.0 (230.0–710.2)	1321.0 (929.0–1784.0)	702.0 (423.0–2080.0)	<0.0001	NA
IL-6 (pg/ml)	1.66 (1.125–2.5)	2.02 (1.84–6.11)	12.48 (5.63–24.5)	27.86 (9.5–63.79)	40.1 (14.3–51.3)	90.42 (34.6–267.3)	<0.0001	NA
BMI (kg/m^2^)	NA	NA	NA	NA	NA	NA	NA	26.2 (22.8–32.5)
120′ plasma glucose during 75 g OGTT	NA	NA	NA	NA	NA	NA	NA	5.9 (5.3–7.4)
HbA1c (% / mmol/mol)	NA	NA	NA	NA	NA	NA	NA	5.5 (5.2–5.6)

Data are median (IQR), n (%) or n/N (%), where N is the total number of patients with available dat a.

Troponin values were not assessed because the two participating institutions were routinely measuring different types of troponins: highly sensitive cardiac troponin T vs I.

p-values are for multiple group comparisons of COVID-19 groups and were calculated using the Kruskal–Wallis test (continuous parameters) or using the multinomial logistic regression mode (nominal variables).

† The term ‘WHO scale’ refers to the peak COVID-19 disease severity on the WHO ordinary scale [12].

‡ All participants were females in the “non-COVID-19” group and underwent 75 g OGTT and sampling prior to September 2019 due to their participation in another study.

§ Diabetes and prediabetes (13/39) were diagnosed based on either the 75 g OGTT results (IFG and/or IGT) or based on the HbA1c or on both criteria. 75 g OGTT and HbA_1c_ data are only available in the ‘non-COVID-19’ control group.

ALAT: Alanine aminotransferase; ASAT: Aspartate aminotransferase; CRP: C-reactive protein; gGT: Gamma glutamyl-transferase; IFG: Impaired fasting glycemia; IGT: Impaired glucose tolerance; IQR: Interquartile range; NA: Not applicable; OGTT: Oral glucose tolerance test; PCT: Procalcitonine.

### Circulating DPP4 activity results

The assumption of normal data distribution of circulating DPP4 activity values in the entire study population was rejected based on the Shapiro–Wilk test (W: 0.9805; p = 0.0119).

**Figure 2.**
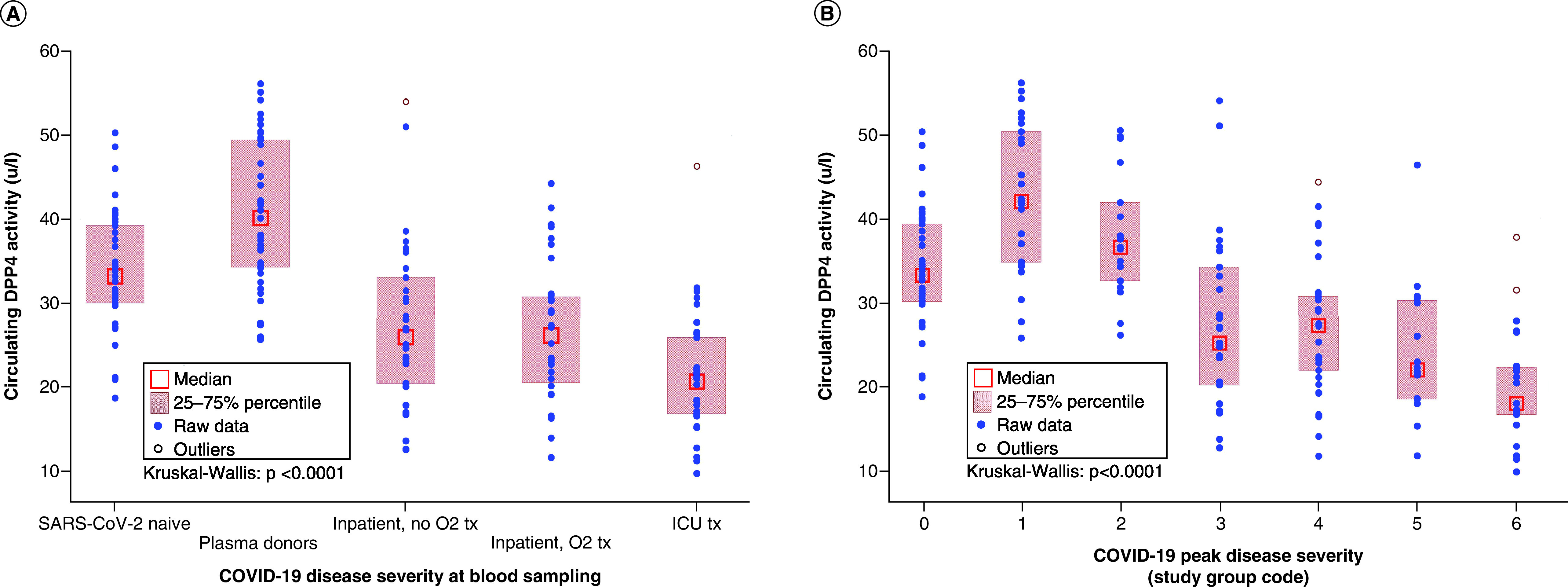
Circulating DPP4 activities stratified by disease severity and outcome categories. **(A)** Circulating DPP4 activity in different COVID-19 disease severity groups at blood sampling. **(B)** Circulating DPP4 activity in different COVID-19 peak disease severity (‘outcome’) groups (study groups as described in the text). Median: small boxes and Q1–Q3: large boxes are indicated.

Therefore, the authors report both the median circulating DPP4 activity and 25th–75th percentile range (Q1–Q3) in the descriptive statistics. These results are stratified by the study groups established based on the disease severity categories, both at the time of blood sampling and at maximum severity during the course of COVID-19 (Supplementary Table 1A & B & Figure 2A & B). The statistical analysis was performed with both approaches.

The authors recognized that the DPP4 activities were different among the different severity categories, both at sampling and at peak severity. The circulating DPP4 activity at blood sampling was decreased (p < 10^-6^) in hospitalized patients with COVID-19 (median: 23.25 U/l; Q1–Q3: 17.80–30.34 U/l) compared with non-acutely ill patients (median: 35.62 U/l;, Q1–Q3: 31.17–42.09 U/l).

The serum DPP4 activities in the samples obtained from patients with acute COVID-19 disease (study group codes: 3-4-5-6) decreased gradually and concurrently with increasing degree of disease severity, ending with the lowest median in the group of those who subsequently died during the hospital stay. The Jonckheere–Terpstra (J-T) trend test reached significance (p = 0.0012) with the test statistics: 1315.500, standard error = 161.373, z statistic = -3.235. This confirmed a significant, gradual decrease in circulating DPP4 activity concurrently with worsening disease severity.

**Figure 3.**
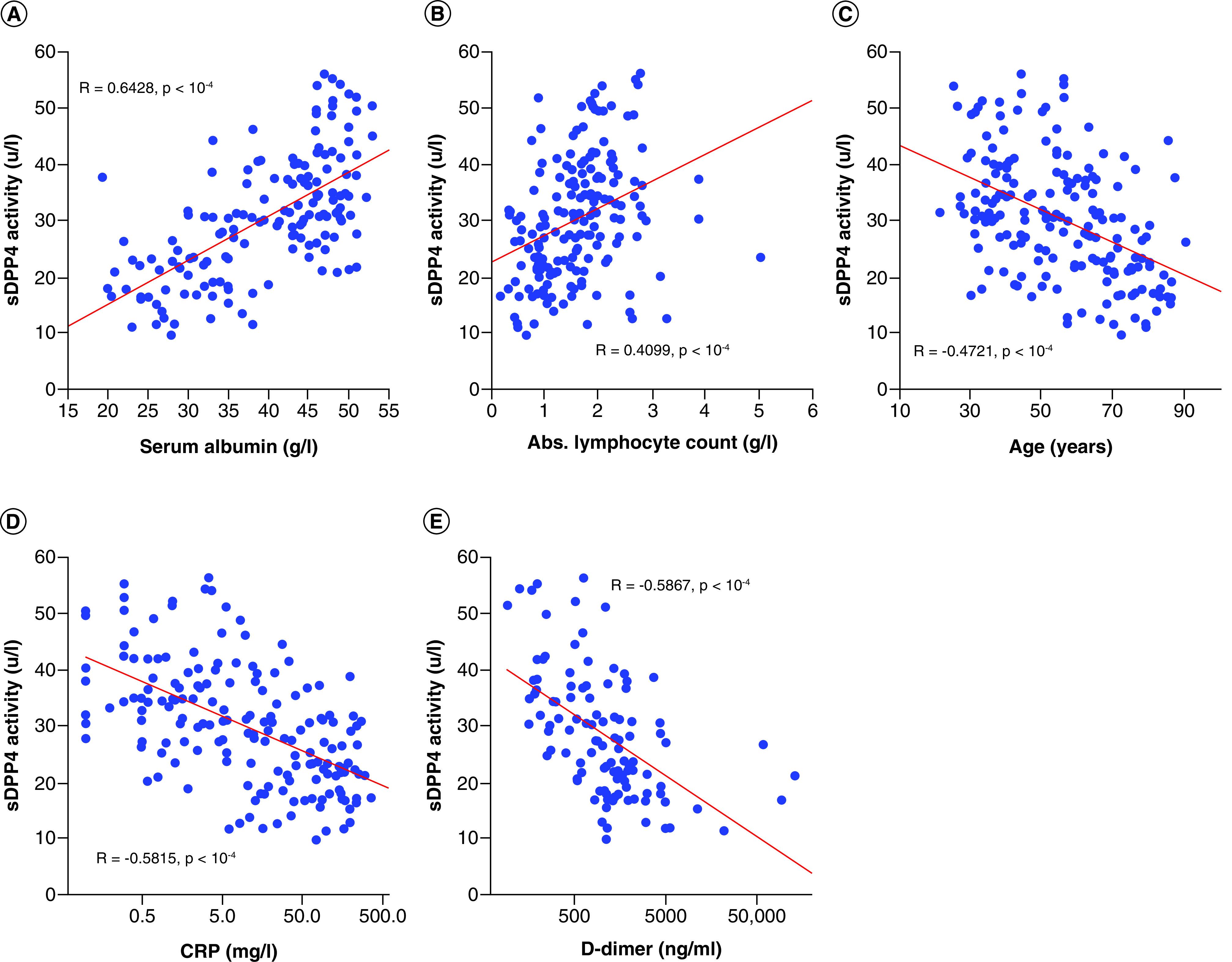
Correlations among serum DPP4 activity and known prognostic factors of COVID-19 disease mortality. **(A)** DPP4 versus albumin (R: 0.6428; p < 0.0001). **(B)** DPP4 versus absolute lymphocyte count (R: 0.4099; p < 0.0001). **(C)** DPP4 versus age (R: -0.4721; p < 0.0001). **(D)** DPP4 versus C-reactive protein (CRP) (R: -0.5815; p < 0.0001). **(E)** DPP4 versus D-dimer (R: -0.5867; p < 0.0001). The axis for CRP and D-dimer are logarithmically scaled and fitted.

The authors found significant correlations among serum DPP4 activity and known prognostic markers of COVID-19 disease severity, such as the patients' ages, absolute lymphocyte count, serum albumin, C-reactive protein (CRP), IL-6 and plasma D-dimer (all p < 0.0001, Figure 3) using the SRO test.

In a logistic regression model, a 1 unit increase of the circulating DPP4 activity was associated with an odds ratio (OR) of 0.85 (b: -0.158; p < 0.0001) for an acute COVID-19 infection (WHO ordinal scale: 3–8) in the combined study population (Table 2).

**Table 2.** Logistic regression models for the probability of acute SARS-CoV-2 infection and death of inpatients with COVID-19.

Logistic regression model	Estimate	Standard error	Wald stat.	Lower CL 95.0%	Upper CL 95.0%	p-value
I. Univariate logistic regression model for acute COVID-19 as outcome						
Intercept	5.067	0.773	43.00	3.55	6.58	<0.0001
sDPP4 activity (U/l)	-0.158	0.024	42.32	-0.21	-0.11	<0.0001
II. Univariate logistic regression model for death as outcome in COVID-19						
Intercept	1.4819	0.837	3.134	-0.159	3.123	0.0767
sDPP4 activity (U/l)	-0.1160	0.0380	9.322	-0.191	-0.042	0.0023
III. Multivariate logistic regression model^†^ for death as outcome in COVID-19						
Intercept	-5.3327	2.2481	5.62681	-9.73897	-0.9265	0.0177
Age (years)	0.0920	0.0282	10.62594	0.03669	0.1473	0.0011
sDPP4 activity (U/l)	-0.0975	0.0394	6.12794	-0.17474	-0.0203	0.0133

I. Model outcome: acute SARS-CoV-2 infection *(WHO ordinal scale: 3–8) versus plasma donor recovered from COVID-19 and ‘before-COVID-19 pandemic’ control groups combined.

II. Model outcome: death versus survival in hospitalized acute COVID-19 patients.

The univariate logistic regression models for 19 other relevant predictor candidates are indicated in Supplementary Table 1.

III. Model outcome: death versus survival in hospitalized acute COVID-19 patients.

† Additional adjustments (13 laboratory predictors and 5 established clinical risk factors) on the relationship between sDPP4 activity and mortality are reported in Supplementary Table 2.

CL: Confidence limit; sDPP4: Soluble DPP4.

A multinomial logit model was used to assess the predictive capacity of circulating DPP4 activity (both as a single biomarker and after adjustment to age) in hospitalized patients with acute SARS-CoV-2 infection using the peak disease severity subgroup as outcome: DPP4 activity: Wald statistics: 10.5225 and p = 0.0146 and DPP4 activity with age: Wald statistics: 8.0988/19.5979 and p = 0.0440/0.0002, respectively.

The association between circulating DPP4 activity and COVID-19 disease mortality in hospitalized patients with acute SARS-CoV-2 infection was also assessed (death as the dependent binary variable and DPP4 as a single prognostic variable) and the logit probability of death was significant for the DPP4 effect (Table 2, corresponding OR of 0.890 for death for every unit increase in serum DPP4 activity).

The relationship between circulating DPP4 activity and mortality in COVID-19 was further adjusted to control for the confounding effects in 19 separate bivariate logistic regressions with the following covariates: age, peripheral blood absolute lymphocyte count, plasma fibrinogen, D-dimer, glucose and serum albumin, aspartate aminotransferase (ASAT), alanine aminotransferase (ALAT), alkaline phosphatase (ALP), CRP, procalcitonin, creatinine, IL-6 and ferritin (troponin could not be used because types were different according to institution) and for previously reported categorical risk factors: hypertension, diabetes mellitus (any type), chronic heart disease, chronic pulmonary disease and malignant disease. When the authors defined a covariate as having a confounder effect provided that the change in p-value (for association between DPP4 activity and mortality in COVID-19) was at least 1 log, then only the plasma glucose and serum albumin concentrations were identified as confounding factors. However, the DPP4 activity effect on mortality remained significant after all the 19 separate adjustments (age, 13 laboratory parameters and five clinical risk factor covariates) and the 95% CI of the estimates remained consistent with the univariate model effect in each case (Supplementary Tables 2 & 3).

**Figure 4.**
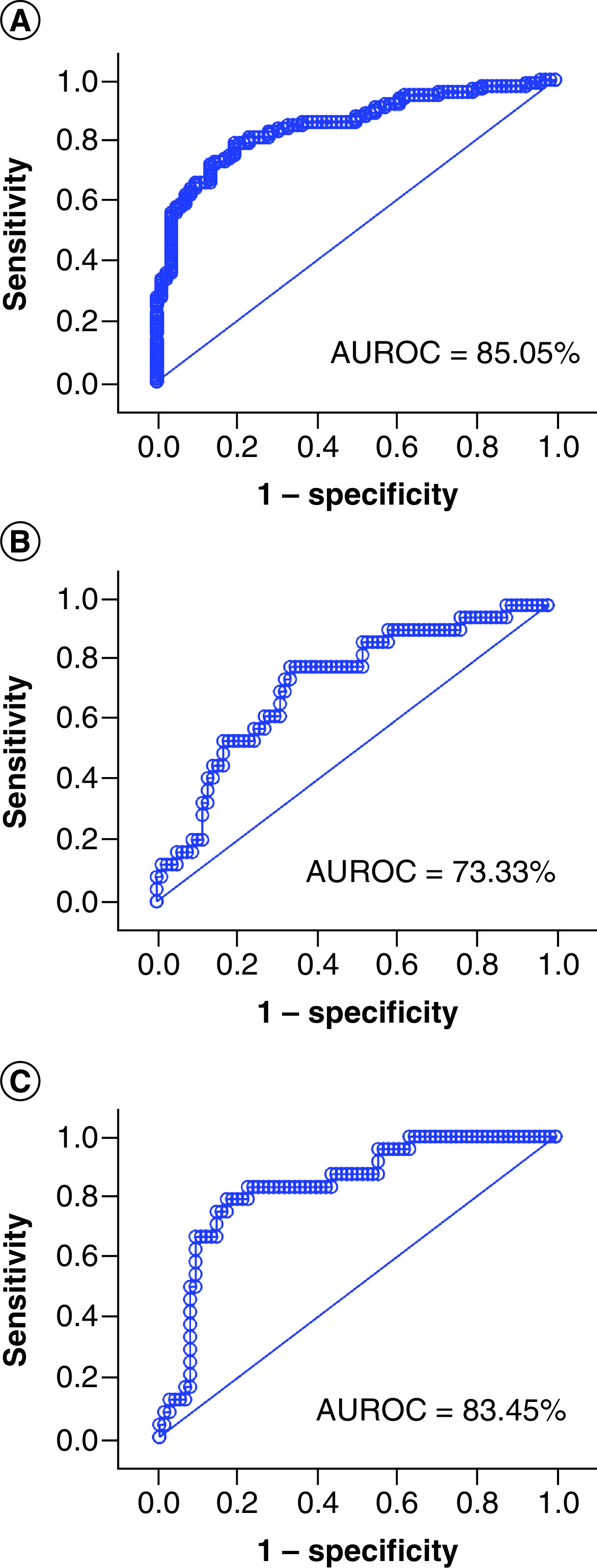
Receiver operating characteristic curves. **(A)** Circulating DPP4 as a single prognostic biomarker for acute SARS-CoV-2 infection (WHO ordinal scale: 3–8), area under ROC (AUROC): 85.05% (95% CI [DeLong]: 79.5–90.61%; p_[area = 0.5]_ < 0.0001; power: >99.99%). **(B)** Circulating DPP4 as a single prognostic biomarker for mortality among patients hospitalized and with acute SARS-CoV-2 infection. AUROC: 73.33% (95% CI: 61.98–84.68%; p_[area = 0.5]_ < 0.0001; power: 94.80%). **(C)** Circulating DPP4 and age as prognostic factors of mortality among patients hospitalized and with acute SARS-CoV-2 infection in a multivariate model. AUROC: 83.45% (95% CI: 74.48–92.51%; p_[area = 0.5]_ < 0.0001; power: 99.98%).

The ROC curve was used to assess the diagnostic ability of circulating DPP4 activity determination as a test to identify individuals with acute SARS-CoV-2 infection (WHO ordinal scale: 3–8) within the entire study group. The sensitivity and specificity were 81.00% and 74.39%, respectively, using a DPP4 activity cutoff value (Youden) of 31.27 U/l with an AUROC of 85.05% (p_[area = 0.5]_ < 0.0001) (Figure 4A). The prognostic ability of serum DPP4 activity as a single biomarker (Figure 4B) and in combination with age (Figure 4C) in predicting the probability of death among hospitalized patients with acute COVID-19 disease and with median time from sampling to death of 9.5 days was also assessed: sensitivity of 79.17% and specificity of 65.8% (DPP4 activity cutoff: 22.25 U/l) with an AUROC of 73.33% (p_[area = 0.5]_ < 0.0001) and sensitivity of 79.2% and specificity of 82.9% with an AUROC of 83.45% (p_[area = 0.5]_ < 0.0001), respectively.

## Discussion

To the authors' knowledge, this is the first report about the alterations in circulating DPP4 enzymatic activity in the serum of patients with and recovered from acute COVID-19 disease. They found that circulating DPP4 activity is decreased in the serum of hospitalized patients with acute COVID-19 in comparison with that of patients recovered from acute COVID-19 or those who were never exposed to SARS-CoV-2. The reduction in serum DPP4 activity among hospitalized patients with acute COVID-19 was associated with increasing clinical severity and was lowest in the group of patients who subsequently died. This sound pattern of alteration in enzymatic activity indicates a strong relationship between DPP4 activity and the clinical course and mortality of COVID-19 disease. Serum DPP4 activity as a biomarker is characterized with competitive test attributes among the recently proposed tests in COVID-19 [21,22,23], in particular with the currently reported performance measures, including ROC curves.

Bioinformatic approaches proposed that there might be a high affinity between human DPP4 and the spike (S1) RBD of SARS-CoV-2 [4,5]. Flexible molecular docking simulations also predicted interactions between the RBD of SARS-CoV-2 and DPP4; however, the interactions were predicted to be weaker than with MERS-CoV [6].

However, subsequent *in vitro* experiments reported that, unlike MERS-CoV, where DPP4 served as a functional receptor (human coronavirus Erasmus Medical Center [hCoV-EMC, later named MERS]) [24], SARS-CoV-2 does not directly bind human DPP4 [7]. In T2DM, which is an established risk factor for COVID-19 disease course, the circulating DPP4 activity was found to be significantly increased in prior reports [25,26]. Consistently, a few authors suggested, based on these theoretical points regarding DPP4 activity in at-risk medical conditions, that the increased circulating levels of soluble DPP4 should contribute to the severity of COVID-19 [27].

On the other hand, a recent study reported reduced soluble CD26 (DPP4) protein levels in age-related dementia (ARD) in older people and in patients with T2DM [28]. ARD and advanced age are considered risk factors for susceptibility to SARS-CoV-2 infection [28]. Recently, reduced protein levels of DPP4 in human subjects hospitalized with COVID-19 infections in comparison with healthy human subjects were also reported [29,30]. However, the sample size in the first study was particularly low [29], and despite that the latter study found a similar gradual decrease in DPP4 protein concentration with increasing disease severity [30] none of these studies reported any association with COVID-19 mortality. In addition, it was previously reported that DPP4 serum protein concentrations significantly diverge from DPP4 enzymatic activities in many pathologies, including autoimmune diseases [31], obesity [32] and experimentally increased oxidative stress conditions [33], and hypoxia induced a decrease of the released DPP4 activity [34]. Many of these conditions are relevant factors in the disease course of COVID-19. Furthermore, the authors reveal prior significant, unreported correlations between circulating DPP4 activity and CRP, D-dimer, IL-6 and peripheral blood lymphocyte count, which are all essential parameters currently used in the everyday clinical care of COVID-19 patients. These latter findings may underline that the enzymatic activity is a different, but at least equally important, biological property than the protein concentration of a molecule with measurable enzymatic activity, such as the soluble DPP4. The significant correlation the authors found between serum DPP4 activity and absolute lymphocyte count in combination with prior experimental findings may suggest that a large proportion of circulating DPP4 could originate from lymphocytes [35]. The correlations between circulating DPP4 activity and albumin and patients' ages, from the clinical perspective, are also consistent with prior reports [28,36]. The association between decreased serum DPP4 activity and peak COVID-19 severity outcomes is also consistent with the prior report indicating that serum DPP4 activity is decreased in severe sepsis [37].

The authors could only identify the plasma glucose and serum albumin levels as confounding factors (resulting in at least a 1 log change of the p for the relationship between circulating DPP4 activity and mortality); however, the DPP4 activity remained significant as a predictor in the model of mortality even after adjustment to these confounders. The latter in combination with the finding that circulating DPP4 activity could serve as a negative inflammatory biomarker in acute COVID-19 disease that inversely correlates with CRP and directly correlates with a major negative acute-phase protein albumin level [36] is a novel understanding. The role of plasma glucose level as a confounding factor with a significant effect on the strong relationship between serum DPP4 activity and mortality in COVID-19 might, in theory, be due to very different pathophysiologic explanations – for example, an altered metabolic dysregulation in the DPP4–incretin axis or alternatively a viral inflammation-related increase in insulin resistance with subsequent hyperglycemia [38] that simply occurs in parallel with a reduction in circulating DPP4 activity.

The beneficial effect of DPP4 inhibitors on COVID-19 outcomes was raised; however, only retrospective analyses are available and not all of these reports consistently demonstrated a beneficial effect [9,10,39,40,41,42,43,44]; the results from prospective trials are lacking. In theory, different mechanisms could explain why this drug class effect was assessed, such as the immunomodulatory effect [45] (both via direct DPP4 inhibition [45] and via GLP-1 [46]), the potentially shorter time in which T2DM patients spent in hypoglycemia [47], lower insulin need and the possibility of interference with a weak DPP4–virus binding interaction [6]. DPP4 was also proposed as an adipokine hormone [48] and it was reported that circulating DPP4 activity is increased in patients with NAFLD [14,49]. This in combination with the facts that both obesity [13] and fatty liver disease (even after adjustment to BMI) [50,51] are known risk factors for severe COVID-19 disease makes it highly unlikely that the decrease in circulating DPP4 activity in severe COVID-19 could be explained by the higher BMI values.

This outlines that the role of further speculation on the decreased serum DPP4 activity in COVID-19 pathology remains limited without a deeper understanding of a clear chain of causality. In addition, the inverse correlation between DPP4 activity and D-dimer levels also urges further experimental research in particular, due to the fact that the increased D-dimer levels and thromboembolic events observed during the COVID-19 disease course and are associated with short-term mortality [21,52].

Interestingly, genetic findings suggested that a risk haplotype in the extended promoter region of the *DPP4* gene inherited from Neandertals increased the risk of critical illness in COVID-19 [8], and the rs3788979 *DPP4* intron variant was associated with COVID-19 as well as with lower serum DPP4 protein concentration; however, the enzymatic activity was not reported [30].

The binding and interaction between SARS-CoV-2 and DPP4 proposed earlier could have explained, in theory, the reduction in DPP4 activity [4,5,6]; however, direct binding was reported to be excluded by experiments using a purified soluble recombinant human DPP4 with appropriate enzymatic activity [7]. Nevertheless, it is interesting that the prediction of the SARS-CoV-2 binding sites on DPP4 was modeled on a DPP4 monomer protein structure [4,5,6] and the monomeric DPP4 is enzymatically inactive [53], in contrast to the dimeric and tetrameric forms that are active. Therefore, in theory, one may cautiously hypothesize that the altered dimerization might also play a role in the explanation for the decreased DPP4 enzymatic activity reported here.

Other explanations, such as the increased oxidative stress, or role of *DPP4* gene variants, or altered regulation of DPP4 mRNA, or protein expression, or decreased release of the soluble form into the serum should all be considered only as possible but not yet fully proven mechanisms to explain the reduced DPP4 serum activity in COVID-19 disease.

## Conclusion

The authors concluded that serum circulating DPP4 activity is a strong biomarker of mortality, and this effect remains significant after adjustments for 13 relevant laboratory parameters and five clinical risk factor covariates. As a single biomarker (using the analysis defined cutoff), it could identify those cases with a sensitivity of nearly 80% who died a median 9.5 days after the sampling, which refers to its potential everyday utility in clinical care. It may be stated that circulating DPP4 activity determination is an uncomplicated *in vitro* method that might aid in the rapid, accurate and early prediction of COVID-19 disease progression, the determination of related therapeutic treatment needs and clinical decisions about the level of medical care required. Additional studies are needed to clarify that this first reported alteration in serum DPP4 activity in COVID-19 is limited only to the original SARS-CoV-2 variant or it also characterizes COVID-19 caused by other virus variants and whether it may help the patient management in the ongoing pandemic .

### Limitations

The sampling in this study was not ultimately performed at the admission of hospitalized patients with COVID-19 but during their hospital stay. In addition, outpatients with acute SARS-CoV-2 infection were not enrolled, and this makes the conclusions formally valid only for hospitalized patients with acute COVID-19; however, the significant trend in circulating DPP4 activity reduction associated consistently with more severe COVID-19 outcomes might attenuate these limitations. Although the authors had access to good-quality medical records of hospitalized patients, a few important parameters could not be assessed, such as BMI and troponin levels, due to patient care priorities or institutional differences, respectively. There are no nationally or internationally accepted reference levels of circulating DPP4 activity on automated laboratory platforms; therefore, the reported biomarker test characteristics were based on ROC curve analyses and Q1–Q3s of the corresponding manual measurements.

**Summary points**

- Type 2 diabetes mellitus and obesity are major risk factors for COVID-19 disease course and mortality.
- Retrospective observational studies previously reported improvement among patients with Type 2 diabetes mellitus using drugs specifically designed to inhibit DPP4 activity and COVID-19 in severe outcomes and mortality.
- Serum DPP4 activity was assessed in a total of 184 individuals, including 102 hospitalized patients with COVID-19, 43 post-COVID-19 plasma donors and 39 individuals who were never exposed to SARS-CoV-2 (with sampling prior to the pandemic).
- Circulating DPP4 enzyme activity was lower in the serum of hospitalized patients with ongoing acute SARS-CoV-2 infection compared with both plasma donors who had already recovered from acute COVID-19 and those who were never exposed to the virus.
- A significant, gradual decrease in circulating DPP4 activity occurred concurrently with worsening disease severity among hospitalized COVID-19 patients (none of them on DPP4 inhibitor), and the lowest DPP4 values occurred in the group of those who subsequently died during their hospital stay.
- Circulating DPP4 activity is a predictor of mortality (median time from sampling to death: 9.5 days).
- Serum DPP4 activity significantly correlated with clinically meaningful parameters in COVID-19, including the patients' ages, absolute lymphocyte count and serum albumin, C-reactive protein, IL-6 and plasma D-dimer levels.
- The effect of DPP4 activity on COVID-19 mortality remained significant even after adjustments to 19 established risk factor covariates.
- Serum DPP4 activity is associated with COVID-19 severity and is a strong prognostic biomarker of mortality.
