# Supplementary material for: Decreased circulating dipeptidyl peptidase-4 enzyme activity is prognostic for severe outcomes in COVID-19 inpatients
Source: Biomark Med. 2022 Feb 23;16(5):317–30. doi: 10.2217/bmm-2021-0717 (PMC8961475; doi:10.2217/bmm-2021-0717)
Supplement: Supplementary file 1 [file bmm-16-317-s1.docx]

**Title: Decreased circulating dipeptidyl-peptidase 4 enzyme activity is prognostic for severe outcomes in COVID-19 inpatients**

**Supplementary Material:**

Table S1 Descriptive statistics and Kruskal-Wallis ANOVA by ranks of DPP4 serum activity in different groups stratified by SARS-CoV-2 infection status and the COVID-19 disease outcome (A: disease severity at serum sampling; B: maximal severity)

Table S2 Univariate logistic regression models for the assessment of the relationship between 20 candidate predictor variables and inpatient mortality in COVID-19

Table S3 Multivariate logistic regression models after adjustment the cDPP4 activity effect to 19 reported risk factors as covariates to assess the probability of inpatient death in COVID-19

# **Table S1**

## **A: Descriptive statistics and Kruskal-Wallis ANOVA by ranks of DPP4 serum activity in different groups stratified by SARS-CoV-2 infection status and the COVID-19 disease outcome (disease severity at serum sampling)**

| **Study group** | **Valid n** | **Mean** | **(SD)** | **Median** | **(Q1-Q3)** | **Sum of Ranks** | **Mean Rank** |
| --- | --- | --- | --- | --- | --- | --- | --- |
| SARS-CoV-2 naive | 39 | 33.83 | (7.08) | 33.20 | (30.01-39.27) | 4353.00 | 111.62 |
| Plasma donors | 43 | 40.55 | (8.56) | 40.16 | (34.28-49.49) | 6024.00 | 140.09 |
| Inpatient, no O_2_ tx | 30 | 27.31 | (10.04) | 25.89 | (20.36-33.06) | 2252.00 | 75.07 |
| Inpatient, O_2_ tx | 36 | 26.25 | (8.44) | 26.10 | (20.49-30.76) | 2548.50 | 70.79 |
| ICU tx | 34 | 21.12 | (7.31) | 20.60 | (16.73-25.84) | 1475.50 | 43.40 |
| **Kruskal-Wallis test: H (4, N= 182) =79.09 p <0.0001**  **Abbreviation: DPP4, dipeptidyl-peptidase 4; SD, standard deviation; Q1-Q3, 25^th^-75^th^ percentile range** | | | | | | | |

## **B: Descriptive statistics and Kruskal-Wallis ANOVA by ranks of DPP4 serum activity in different groups stratified by SARS-CoV-2 infection status and the COVID-19 disease outcome (maximal severity)***

| **Study group code** | **Valid n** | **Mean** | **(SD)** | **Median** | **(Q1-Q3)** | **Sum of Ranks** | **Mean Rank** |
| --- | --- | --- | --- | --- | --- | --- | --- |
| 0 | 39 | 33.83 | (7.08) | 33.20 | (30.01-39.27) | 4353.00 | 111.62 |
| 1 | 26 | 42.27 | (8.85) | 41.98 | (34.72-50.34) | 3796.00 | 146.00 |
| 2 | 17 | 37.91 | (7.61) | 36.54 | (32.50-41.88) | 2228.00 | 131.06 |
| 3 | 27 | 27.25 | (10.53) | 25.02 | (19.99-34.13) | 2020.00 | 74.82 |
| 4 | 33 | 26.91 | (7.96) | 27.10 | (21.75-30.58) | 2436.00 | 73.82 |
| 5 | 16 | 24.05 | (8.36) | 21.80 | (18.32-30.15) | 925.00 | 57.81 |
| 6 | 24 | 19.75 | (6.63) | 17.80 | (16.47-22.12) | 895.00 | 37.29 |
| **Kruskal-Wallis test: H (6, N= 182) =81.47, p<0.0001**  ***Study groups and corresponding peak disease severities on the WHO ordinal scale are defined in the text**  **Abbreviation: DPP4, dipeptidyl-peptidase 4; SD, standard deviation; Q1-Q3, 25^th^-75^th^ percentile range**  Study group codes:  0: SARS-CoV-2 naïve control group (samples from a female population before September 2019)  1: no prior hospitalisation due to COVID-19  2: prior hospitalisation due to COVID-19  3: not requiring O2 therapy (WHO ordinal scale: 3)  4: required O2 therapy via nasal cannula only (WHO ordinal scale: 4)  5: intensive care unit (ICU) admission required (WHO ordinal scale 6+7)  6: died in COVID-19 (WHO ordinal scale: 8) | | | | | | | |

# **Table S2 Univariate models** **for the probability of inpatient death due to COVID-19**

|  | **Estimate** | **Standard Error** | **Wald Stat.** | **Lower CL 95.0%** | **Upper CL 95.0%** | **crude p** |
| --- | --- | --- | --- | --- | --- | --- |
|  |  |  |  |  |  |  |
| **cDPP4 activity** | | | | | | |
| Intercept | 1.4820 | 0.8372 | 3.1337 | -0.1588 | 3.1228 | 0.0767 |
| cDPP4 activity (U/L) | -0.1160 | 0.0380 | 9.3222 | -0.1904 | -0.0415 | 0.0023 |
|  |  |  |  |  |  |  |
| **Age** | | | | | | |
| Intercept | -8.2483 | 2.0058 | 16.9101 | -12.1797 | -4.3170 | <0.0001 |
| Age (years) | 0.1026 | 0.0274 | 14.0414 | 0.0489 | 0.1563 | 0.0002 |
|  |  |  |  |  |  |  |
| **Absolute lymphocyte count** | | | | | | |
| Intercept | 0.7253 | 0.5698 | 1.6200 | -0.3916 | 1.8421 | 0.2031 |
| Absolute lymphocyte count (G/L) | -1.6245 | 0.5218 | 9.6912 | -2.6473 | -0.6017 | 0.0019 |
|  |  |  |  |  |  |  |
| **Plasma fibrinogen level** | | | | | | |
| Intercept | 0.5371 | 0.9382 | 0.3277 | -1.3017 | 2.3759 | 0.5670 |
| Fibrinogén (g/L) | -0.2127 | 0.1642 | 1.6770 | -0.5346 | 0.1092 | 0.1953 |
|  |  |  |  |  |  |  |
| **D-dimer level** | | | | | | |
| Intercept | -1.3158 | 0.3163 | 17.3055 | -1.9357 | -0.6958 | <0.0001 |
| D-dimer (ng/mL) | 0.0001 | 0.0001 | 2.0685 | -0.0001 | 0.0003 | 0.1504 |
|  |  |  |  |  |  |  |
| **Plasma glucose level** | | | | | | |
| Intercept | -3.4389 | 1.0423 | 10.8847 | -5.4818 | -1.3959 | 0.0010 |
| Plasma glucose (mmol/L) | 0.3338 | 0.1707 | 3.8226 | -0.0008 | 0.6684 | 0.0506 |
|  |  |  |  |  |  |  |
| **Serum aspartate aminotransferase level** | | | | | | |
| Intercept | -2.1813 | 0.4659 | 21.9239 | -3.0944 | -1.2683 | <0.0001 |
| ASAT (U/L) | 0.0224 | 0.0087 | 6.5990 | 0.0053 | 0.0394 | 0.0102 |
|  |  |  |  |  |  |  |
| **Serum alanin aminotransferase level** | | | | | | |
| Intercept | -1.3379 | 0.2744 | 23.7729 | -1.8758 | -0.8001 | <0.0001 |
| ALAT (U/L) | 0.0041 | 0.0030 | 1.8527 | -0.0018 | 0.0100 | 0.1735 |
|  |  |  |  |  |  |  |
| **Serum creatinine level** | | | | | | |
| Intercept | -2.0018 | 0.4104 | 23.7864 | -2.8062 | -1.1973 | <0.0001 |
| Creatinin (µmol/L) | 0.0080 | 0.0030 | 7.1032 | 0.0021 | 0.0139 | 0.0077 |
|  |  |  |  |  |  |  |
| **Serum albumin level** | | | | | | |
| Intercept | 1.3630 | 1.3069 | 1.0877 | -1.1985 | 3.9246 | 0.2970 |
| Albumin (g/L) | -0.0759 | 0.0414 | 3.3706 | -0.1570 | 0.0051 | 0.0664 |
|  |  |  |  |  |  |  |
| **Serum C reactive protein level** | | | | | | |
| Intercept | -2.0767 | 0.3997 | 26.9970 | -2.8600 | -1.2933 | <0.0001 |
| CRP (mg/L) | 0.0100 | 0.0029 | 11.5704 | 0.0042 | 0.0157 | 0.0007 |
|  |  |  |  |  |  |  |
| **Serum procalcitonin level** | | | | | | |
| Intercept | -1.0505 | 0.2342 | 20.1170 | -1.5095 | -0.5914 | <0.0001 |
| PCT (ng/mL) | -0.0052 | 0.0247 | 0.0450 | -0.0537 | 0.0432 | 0.8320 |
|  |  |  |  |  |  |  |
| **Serum IL-6 level** | | | | | | |
| Intercept | -1.4692 | 0.2711 | 29.3653 | -2.0006 | -0.9378 | <0.0001 |
| IL-6 (pg/mL) | 0.0019 | 0.0009 | 4.3488 | 0.0001 | 0.0037 | 0.0370 |
|  |  |  |  |  |  |  |
| **Serum ferritin level** | | | | | | |
| Intercept | -1.4116 | 0.3241 | 18.9747 | -2.0467 | -0.7764 | <0.0001 |
| Ferritin (ng/mL) | 0.0004 | 0.0002 | 2.4974 | -0.0001 | 0.0008 | 0.1140 |
|  |  |  |  |  |  |  |
| **Serum alkaline phosphatase level** | | | | | | |
| Intercept | -1.7418 | 0.4436 | 15.4150 | -2.6113 | -0.8723 | 0.0001 |
| ALP (U/L) | 0.0066 | 0.0034 | 3.6772 | -0.0002 | 0.0133 | 0.0552 |
|  |  |  |  |  |  |  |
| **Hypertension** |  |  |  |  |  |  |
| Intercept | -1.3287 | 0.2757 | 23.2308 | -1.8691 | -0.7884 | <0.0001 |
| Hypertension | 0.4958 | 0.2757 | 3.2347 | -0.0445 | 1.0361 | 0.0721 |
|  |  |  |  |  |  |  |
| **Diabetes mellitus** |  |  |  |  |  |  |
| Intercept | -0.8726 | 0.2498 | 12.2011 | -1.3623 | -0.3830 | 0.0005 |
| Diabetes mellitus (any type) | 0.6315 | 0.2498 | 6.3890 | 0.1418 | 1.1211 | 0.0115 |
|  |  |  |  |  |  |  |
| **Chronic heart disease** | | | | | | |
| Intercept | -1.0438 | 0.2368 | 19.4253 | -1.5079 | -0.5796 | <0.0001 |
| Chronic heart disease | 0.3062 | 0.2368 | 1.6714 | -0.1580 | 0.7703 | 0.1961 |
|  |  |  |  |  |  |  |
| **Chronic pulmonary disease** | | | | | | |
| Intercept | -0.8770 | 0.2580 | 11.5592 | -1.3826 | -0.3714 | 0.0007 |
| Chronic pulmonary disease | 0.5093 | 0.2580 | 3.8980 | 0.0037 | 1.0149 | 0.0483 |
|  |  |  |  |  |  |  |
| **Malignant disease** | | | | | | |
| Intercept | -0.8901 | 0.2557 | 12.1210 | -1.3911 | -0.3890 | 0.0005 |
| Malignant disease | 0.4482 | 0.2557 | 3.0739 | -0.0528 | 0.9493 | 0.0796 |
|  |  |  |  |  |  |  |

**Abbreviations: cDPP4, circulating dipeptidyl-peptidase 4; CL, confidence limit**

# **Table S3 Adjusting the effect of cDPP4 activity in multivariate models for the probability of inpatient death due to COVID-19**

|  | **Estimate** | **Standard Error** | **Wald Stat.** | **Lower CI 95.0%** | **Upper CI 95.0%** | **crude p** |
| --- | --- | --- | --- | --- | --- | --- |
|  |  |  |  |  |  |  |
| **cDPP4 activity and age** | | | | | | |
| Intercept | -5.3327 | 2.2481 | 5.6268 | -9.7390 | -0.9265 | 0.0177 |
| cDPP4 activity (U/L) | -0.0975 | 0.0394 | 6.1279 | -0.1747 | -0.0203 | 0.0133 |
| Age (years) | 0.0920 | 0.0282 | 10.6259 | 0.0367 | 0.1473 | 0.0011 |
|  |  |  |  |  |  |  |
| **cDPP4 activity and absolute lymphocyte count** | | | | | | |
| Intercept | 3.4850 | 1.1835 | 8.6702 | 1.1653 | 5.8047 | 0.0032 |
| cDPP4 activity (U/L) | -0.1237 | 0.0436 | 8.0670 | -0.2091 | -0.0384 | 0.0045 |
| Abs. Ly count (G/L) | -1.6296 | 0.5606 | 8.4510 | -2.7282 | -0.5309 | 0.0036 |
|  |  |  |  |  |  |  |
| **cDPP4 activity and plasma fibrinogen level** | | | | | | |
| Intercept | 2.8811 | 1.3155 | 4.7969 | 0.3028 | 5.4594 | 0.0285 |
| cDPP4 activity (U/L) | -0.1095 | 0.0412 | 7.0651 | -0.1903 | -0.0288 | 0.0079 |
| Fibrinogén (g/L) | -0.1942 | 0.1788 | 1.1790 | -0.5446 | 0.1563 | 0.2776 |
|  |  |  |  |  |  |  |
| **cDPP4 activity and plasma D-dimer level** | | | | | | |
| Intercept | 1.5146 | 0.9475 | 2.5553 | -0.3424 | 3.3716 | 0.1099 |
| cDPP4 activity (U/L) | -0.1183 | 0.0419 | 7.9704 | -0.2004 | -0.0362 | 0.0048 |
| D-dimer (ng/mL) | 0.0001 | 0.0001 | 1.6929 | 0.0000 | 0.0002 | 0.1932 |
|  |  |  |  |  |  |  |
| **cDPP4 activity and plasma glucose level** | | | | | | |
| Intercept | -0.5528 | 1.5124 | 0.1336 | -3.5170 | 2.4113 | 0.7147 |
| cDPP4 activity (U/L) | -0.1043 | 0.0459 | 5.1689 | -0.1942 | -0.0144 | 0.0230 |
| Plasma glucose (mmol/L) | 0.2495 | 0.1734 | 2.0711 | -0.0903 | 0.5892 | 0.1501 |
|  |  |  |  |  |  |  |
| **cDPP4 activity and aspartate aminotransferase level (ASAT)** | | | | | | |
| Intercept | 1.2680 | 1.0133 | 1.5659 | -0.7180 | 3.2541 | 0.2108 |
| cDPP4 activity (U/L) | -0.1649 | 0.0497 | 11.0305 | -0.2623 | -0.0676 | 0.0009 |
| ASAT (U/L) | 0.0264 | 0.0094 | 7.8315 | 0.0079 | 0.0448 | 0.0051 |
|  |  |  |  |  |  |  |
| **cDPP4 activity and alanine aminotransferase level (ALAT)** | | | | | | |
| Intercept | 1.2453 | 0.8508 | 2.1423 | -0.4223 | 2.9129 | 0.1433 |
| cDPP4 activity (U/L) | -0.1156 | 0.0392 | 8.6991 | -0.1924 | -0.0388 | 0.0032 |
| ALAT (U/L) | 0.0054 | 0.0042 | 1.6508 | -0.0028 | 0.0137 | 0.1989 |
|  |  |  |  |  |  |  |
| **cDPP4 activity and serum creatinine level** | | | | | | |
| Intercept | 0.4814 | 0.9715 | 0.2456 | -1.4226 | 2.3855 | 0.6202 |
| cDPP4 activity (U/L) | -0.0981 | 0.0388 | 6.3798 | -0.1742 | -0.0220 | 0.0115 |
| Creatinin (µmol/L) | 0.0055 | 0.0030 | 3.3970 | -0.0004 | 0.0113 | 0.0653 |
|  |  |  |  |  |  |  |
| **cDPP4 activity and serum albumin level** | | | | | | |
| Intercept | 2.1928 | 1.4158 | 2.3987 | -0.5822 | 4.9677 | 0.1214 |
| cDPP4 activity (U/L) | -0.0887 | 0.0432 | 4.2206 | -0.1734 | -0.0041 | 0.0399 |
| Albumin (g/L) | -0.0395 | 0.0443 | 0.7941 | -0.1264 | 0.0474 | 0.3729 |
|  |  |  |  |  |  |  |
| **cDPP4 activity and C-reactive protein level (CRP)** | | | | | | |
| Intercept | 0.5369 | 0.9382 | 0.3275 | -1.3019 | 2.3756 | 0.5672 |
| cDPP4 activity (U/L) | -0.1161 | 0.0419 | 7.6691 | -0.1983 | -0.0339 | 0.0056 |
| CRP (mg/L) | 0.0094 | 0.0032 | 8.8181 | 0.0032 | 0.0156 | 0.0030 |
|  |  |  |  |  |  |  |
| **cDPP4 activity and procalcitonin value** | | | | | | |
| Intercept | 1.5381 | 0.8807 | 3.0502 | -0.1880 | 3.2643 | 0.0807 |
| cDPP4 activity (U/L) | -0.1159 | 0.0400 | 8.3913 | -0.1943 | -0.0375 | 0.0038 |
| PCT (ng/mL) | -0.0197 | 0.0280 | 0.4956 | -0.0745 | 0.0351 | 0.4814 |
|  |  |  |  |  |  |  |
| **cDPP4 actvity and IL-6 value** | | | | | | |
| Intercept | 1.2406 | 0.9289 | 1.7839 | -0.5799 | 3.0611 | 0.1817 |
| cDPP4 activity (U/L) | -0.1212 | 0.0426 | 8.0918 | -0.2046 | -0.0377 | 0.0044 |
| IL-6 (pg/mL) | 0.0017 | 0.0009 | 3.8099 | 0.0000 | 0.0034 | 0.0510 |
|  |  |  |  |  |  |  |
| **cDPP4 activity and serum ferritin value** | | | | | | |
| Intercept | 1.0642 | 0.9239 | 1.3267 | -0.7467 | 2.8751 | 0.2494 |
| cDPP4 activity (U/L) | -0.1046 | 0.0391 | 7.1736 | -0.1811 | -0.0281 | 0.0074 |
| Ferritin (ng/mL) | 0.0002 | 0.0002 | 1.1844 | -0.0002 | 0.0006 | 0.2765 |
|  |  |  |  |  |  |  |
| **cDPP4 activity and serum alkaline phosphatase activity** | | | | | | |
| Intercept | 1.0765 | 0.9034 | 1.4199 | -0.6941 | 2.8471 | 0.2334 |
| ALP (U/L) | 0.0094 | 0.0042 | 5.0080 | 0.0012 | 0.0176 | 0.0252 |
| cDPP4 activity (U/L) | -0.1360 | 0.0409 | 11.0598 | -0.2162 | -0.0559 | 0.0009 |
|  |  |  |  |  |  |  |
| **cDPP4 activity and presence of hypertension** | | | | | | |
| Intercept | 1.0529 | 0.8776 | 1.4396 | -0.6670 | 2.7729 | 0.2302 |
| cDPP4 activity (U/L) | -0.1062 | 0.0379 | 7.8614 | -0.1805 | -0.0320 | 0.0051 |
| Hypertension | 0.4693 | 0.3100 | 2.2921 | -0.1383 | 1.0769 | 0.1300 |
|  |  |  |  |  |  |  |
| **cDPP4 activity and presence of diabetes mellitus** | | | | | | |
| Intercept | 1.9129 | 0.9112 | 4.4074 | 0.1270 | 3.6987 | 0.0358 |
| cDPP4 activity (U/L) | -0.1240 | 0.0407 | 9.2979 | -0.2037 | -0.0443 | 0.0023 |
| Diabetes mellitus (any type) | 0.7038 | 0.2757 | 6.5157 | 0.1634 | 1.2443 | 0.0107 |
|  |  |  |  |  |  |  |
| **cDPP4 activity and presence of chronic heart disease** | | | | | | |
| Intercept | 1.4733 | 0.8460 | 3.0328 | -0.1848 | 3.1314 | 0.0816 |
| cDPP4 activity (U/L) | -0.1133 | 0.0384 | 8.7036 | -0.1886 | -0.0380 | 0.0032 |
| Chronic heart disease | 0.2265 | 0.2545 | 0.7917 | -0.2724 | 0.7253 | 0.3736 |
|  |  |  |  |  |  |  |
| **cDPP4 activity and presence of chronic pulmonary disease** | | | | | | |
| Intercept | 1.9298 | 0.9121 | 4.4763 | 0.1421 | 3.7175 | 0.0344 |
| cDPP4 activity (U/L) | -0.1242 | 0.0403 | 9.4830 | -0.2032 | -0.0451 | 0.0021 |
| Chronic pulmonary disease | 0.5919 | 0.2837 | 4.3535 | 0.0359 | 1.1479 | 0.0369 |
|  |  |  |  |  |  |  |
| **cDPP4 activity and presence of malignant disease** | | | | | | |
| Intercept | 1.9308 | 0.9025 | 4.5771 | 0.1619 | 3.6997 | 0.0324 |
| cDPP4 activity (U/L) | -0.1272 | 0.0405 | 9.8734 | -0.2065 | -0.0478 | 0.0017 |
| Malignant disease | 0.3214 | 0.2817 | 1.3018 | -0.2307 | 0.8735 | 0.2539 |

**Abbreviations: cDPP4, circulating dipeptidyl-peptidase 4; CL, confidence limit**
